# Changes in Resting Energy Expenditure in Response to Different Dietary Patterns: A Randomized Clinical Trial Exploratory Sub-Analysis

**DOI:** 10.3390/nu18132053

**Published:** 2026-06-24

**Authors:** Irene García-Gómez, Ainoa Mallorquín Castillo, Cristina Navas-Moreno, José Ignacio Martínez-Montoro, María Molina-Vega, Ana María Gómez-Pérez, Juan Alcaide-Torres, Alba Subiri-Verdugo, María Luisa García-Martín, Isabel Moreno-Indias, Francisco J. Tinahones

**Affiliations:** 1 Department of Endocrinology and Nutrition, Virgen de la Victoria University Hospital, 29010 Málaga, Spain; eneri1203@gmail.com (I.G.-G.); ainoacastillo99@gmail.com (A.M.C.); taty.navas@gmail.com (C.N.-M.); molinavegamaria@gmail.com (M.M.-V.); alba.subiri@ibima.eu (A.S.-V.); isabel.moreno@ibima.eu (I.M.-I.);; 2 IBIMA Plataforma BIONAND, Instituto de Investigación Biomédica de Málaga, 29590 Málaga, Spain; juan.alcaidetorres@gmail.com (J.A.-T.); mlgarcia@ibima.eu (M.L.G.-M.); 3 Faculty of Medicine, University of Málaga, 29071 Málaga, Spain; 4 Centro de Investigación Biomédica en Red-Fisiopatología de La Obesidad y Nutrición (CIBEROBN), Instituto de Salud Carlos III, 28029 Madrid, Spain; 5 Biomedical Magnetic Resonance Laboratory—BMRL, Fundación Pública Andaluza Progreso y Salud (FPS), 41092 Seville, Spain; 6 Biomedical Research Networking Center in Bioengineering, Biomaterials & Nanomedicine (CIBER-BBN), Instituto de Salud Carlos III, 28029 Madrid, Spain

**Keywords:** Mediterranean diet, time-restricted eating, modified alternate-day fasting, calorimetry, resting energy expenditure

## Abstract

Background: Evidence comparing the effects of novel alternative dietary strategies on resting energy expenditure (REE) with a hypocaloric standard Mediterranean diet (MedDiet) with continuous caloric restriction remains limited. This study aimed to evaluate the effects of diets with varying ketogenic potentials—including a very-low-carbohydrate diet (ketogenic diet, KD), time-restricted eating (TRE), and modified alternate-day fasting (mADF)—on the REE of individuals with obesity compared to those of a standard MedDiet. Methods: This was a secondary post hoc sub-analysis of a three-month, parallel-arm, randomized clinical trial (RCT) including 160 adults with obesity (body mass index > 30 kg/m^2^). The participants were randomly assigned to one of five calorie-restricted dietary interventions: control (MedDiet), KD, early time-restricted eating (eTRE), late time-restricted eating (lTRE), or mADF. All interventions featured an individualized energy deficit of 600 kcal/day. In this sub-analysis, a total of 102 participants with valid baseline measures were included. The REE was assessed by indirect calorimetry, and longitudinal trajectories were evaluated using Linear Mixed Models (LMMs) in 98 participants to account for baseline variability and to maximize data retention. Results: The mean age of participants in this sub-analysis was 45.3 years (SD 10.8), and 73.1% were women. The longitudinal modeling confirmed no statistically significant differences in the adjusted REE trajectories among the five dietary groups over the 3-month intervention (Group × Time interaction, *p* = 0.506). Furthermore, the LMMs showed that total body weight (*p* < 0.001) and biological sex (*p* < 0.001) were the variables most strongly associated with REE within the model. No independent associations between circulating beta-hydroxybutyrate levels and REE trajectories were detected. Conclusions: Hypocaloric diets with varying macronutrient distributions and fasting windows did not show statistically significant differences in REE trajectories over the 3-month intervention. In this exploratory sub-analysis, the REE trajectories were more closely associated with individual biological characteristics, particularly body weight and sex, than with the specific dietary strategy employed. Given the modest sample size and exploratory nature of the study, these findings should be interpreted cautiously and require confirmation in larger, adequately powered prospective trials.

## 1. Introduction

Obesity is a major public health problem due to its high prevalence worldwide and its related comorbidities [1,2,3]. Achieving and maintaining weight loss in individuals with obesity remains a significant clinical challenge, largely due to the physiological decline in resting energy expenditure (REE) that occurs during periods of negative energy balance [4]. This process is related to metabolic adaptation, defined as a reduction in REE greater than expected based on changes in body composition, primarily affecting the loss of fat mass (FM) and fat-free mass (FFM). From a physiological [5] and evolutionary standpoint [6], the decrease in REE acts as a compensatory mechanism that contributes to the defense of body weight against prolonged energy restriction. In prospective observational cohorts, a low body mass-adjusted metabolism has been shown to predict long-term weight regain [7]. However, the reduction in REE associated with metabolic adaptation cannot be fully explained by the loss of metabolically active tissues. Several physiological mechanisms have been proposed to explain this phenomenon, including hormonal alterations (especially reductions in leptin and thyroid hormones), changes in sympathetic nervous system activity, modifications in tissue metabolic efficiency, and adjustments in energy substrate utilization. These adaptations reflect an integrated physiological response aimed at improving energy efficiency and conserving energy in an environment perceived as energy deficient [5].

To address rising obesity, and with continuous caloric restriction (CR) as a cornerstone, various dietary strategies have been proposed. Different dietary approaches, such as ketogenic diets (KDs) and intermittent fasting (IF), have gained popularity for their potential metabolic benefits beyond weight loss [8]. Despite the efficacy of these strategies in the short-term, their long-term maintenance may be obstructed by metabolic adaptation (adaptive thermogenesis) [9]. Consequently, the research is shifting focus from macronutrient composition and timing to the specific metabolic pathways stimulated, searching for both weight reduction and enhanced metabolic health. In this context, the evidence regarding metabolic adaptation in different dietary approaches is scarce. A KD is a high-fat, low-carbohydrate alternative to traditional CR that induces ketosis, shifting the body’s primary energy source to fatty acids and ketone bodies. This process fuels the central nervous system while it suppresses appetite and improves insulin sensitivity. Though originally designed for drug-resistant epilepsy, its proven metabolic and neurological benefits have expanded its clinical use to treating obesity, metabolic syndrome, and type 2 diabetes [10]. However, the existence of a sustained “metabolic advantage” with KD remains highly debated, as strictly controlled trials have often reported minimal or inconsistent effects on energy expenditure once rigorous adjustments are made for body composition or energy intake [11]. IF, including patterns like time-restricted eating (TRE) and alternate-day fasting (ADF), triggers adaptive mechanisms like KD. By depleting hepatic glycogen stores, typically after 12–14 h, the body shifts from glucose to fatty acid metabolism, resulting in ketone bodies production [12]. This metabolic switch reduces insulin secretion, lowers systemic inflammation, and activates stress-response pathways [5]. Furthermore, within TRE protocols, the specific timing of the eating window introduces distinct chronobiological effects, as it may align nutrient ingestion with a better peak of insulin sensitivity and metabolic rhythms, potentially modifying neuroendocrine regulation and metabolic efficiency [13,14]. While clinical trials have shown that IF can effectively reduce body weight and improve lipid profiles, its long-term efficacy remains uncertain due to the short study durations and high methodological heterogeneity [8].

There is a substantial lack of solid evidence in the literature regarding the real impact of these dietary interventions on the REE [15] compared with traditional patterns, such as a Mediterranean diet (MedDiet), which remains the most recommended model in clinical guidelines. Moreover, recent evidence suggests that different hypocaloric dietary strategies may exert heterogeneous effects on body composition, particularly regarding the preservation of FFM, which is the main determinant of REE, along with other components, such as age or sex [16]. There is a need to clarify whether KDs and IF, in their different modalities, induce a lower change in the REE that compromises or favors basal metabolism, and whether they offer additional or similar advantages compared with a MedDiet. Even when sharing a comparable caloric deficit, these strategies could theoretically yield different REE trajectories due to distinct metabolic and chronobiological shifts, which may modulate sympathetic tone, thyroid axes, or signaling pathways, compared to traditional CR [17]. Under these premises, the present exploratory secondary sub-analysis of a clinical trial aims to evaluate and compare, using indirect calorimetry, the effects on the REE in adults with obesity of different hypocaloric dietary strategies with varying ketogenic potentials versus continuous CR based on a MedDiet after a three-month intervention.

## 2. Materials and Methods

### 2.1. Study Design

A randomized, prospective, parallel-group clinical trial with a duration of 3 months was conducted at the Department of Endocrinology and Nutrition of the Virgen de la Victoria University Hospital (Málaga, Spain). Participants were assigned in a 1:1:1:1:1 ratio to one of the following dietary intervention groups: Mediterranean diet (MedDiet; control group), ketogenic diet (KD), early time-restricted eating (eTRE), late time-restricted eating (lTRE), or modified alternate-day fasting (mADF). Assessment of REE by indirect calorimetry was performed at baseline and at 3 months (end of intervention) as an exploratory secondary post hoc sub-analysis.

The study protocol was approved by the Research Ethics Committee of Málaga (Approval ID: 1/2019-P14) and was conducted in accordance with the principles of the Declaration of Helsinki. The trial was registered with ClinicalTrials.gov (identifier: NCT04453150). All participants provided written informed consent prior to their inclusion in the study. The primary results of this trial have previously been reported, and further methodological details can be found in prior publications [18,19].

### 2.2. Participants and Randomization

The participants were recruited from the outpatient clinics of the Department of Endocrinology and Nutrition of the Virgen de la Victoria University Hospital. The inclusion criteria were age between 18 and 65 years and body mass index (BMI; calculated as weight in kilograms divided by the square of height in meters) between 30 and 45 kg/m^2^. The exclusion criteria included: diabetes mellitus; current or planned pregnancy or lactation; history of eating disorders; use of drugs with potential effects on body weight; unstable weight (variations >5% in the previous 3 months); history of major cardiovascular events in the 6 months prior to inclusion; active cancer; acute inflammatory or infectious diseases; liver dysfunction; chronic kidney disease; use of probiotics, prebiotics, or antibiotics; and excessive alcohol consumption (>30 g/day in men and >20 g/day in women).

The participants were randomized (1:1:1:1:1) into one of five dietary interventions using a computer-generated sequence with allocation concealment. Randomization was implemented using a fixed block size of five to ensure balanced group sizes. Because the primary endpoints of the parent trial were not specifically focused on REE, the randomization procedure did not include additional a priori stratification by sex, baseline anthropometry or other metabolic characteristics. This sub-analysis included participants from the main trial who had valid indirect calorimetry assessments. Missing calorimetry assessments were administrative in nature, resulting from technical equipment failures or unforeseeable clinical scheduling conflicts. These events were not related to the participants’ adherence to the intervention, weight loss efficacy, or any predefined metabolic outcome. The full trial details are reported elsewhere [18,19].

### 2.3. Dietary Interventions, Outcomes and Follow-Up

At the beginning of the study, the participants received personalized nutritional counseling and plans based on a standardized 600 kcal/day deficit (Harris–Benedict). Five hypocaloric interventions (all with the same level of caloric restriction) were compared: a standard MedDiet, a KD (5% carbohydrates), an eTRE (eating window between 08:00 and 16:00), an lTRE (eating window between 14:00 and 22:00), and an mADF (3 fasting days/week at 25–30% energy). To monitor compliance with caloric restriction and assigned fasting windows, nutritionists assessed adherence continuously during face-to-face visits and through regular interim telephone calls. Additionally, KD compliance was verified via weekly fasting capillary beta-hydroxybutyrate (BHB) level measurements using a ketone meter. All participants received standardized counseling on physical activity, which was uniform for all intervention groups and was also monitored using accelerometers as part of the parent trial’s protocol. Further information regarding the specific dietary compositions, meal replacement protocols and full trial methodology can be found in previous publications [18,19].

The present sub-analysis aimed to analyze differences in the REE from baseline to 3 months among the five intervention groups. Two in-person visits were scheduled to assess the REE (at 0 and 3 months). Additionally, two intermediate in-person visits were performed at 1 and 2 months to monitor weight loss, review dietary adherence and resolve doubts. Clinical and anthropometric data were collected at baseline and at the follow-up visit, including weight (kg), height (cm), and waist and hip circumferences (cm), using standardized methods. Body composition (lean mass and fat mass in kg and percentage) was evaluated by a bioelectrical impedance analysis using a Tanita analyzer (TANITA, Arlington Heights, IL, USA). REE estimation was performed using a FITMATE™ RMR calorimeter (Cosmed Corporation, Rome, Italy) before and after the intervention. The FITMATE ™ RMR calorimeter quantifies ventilation via a turbine flowmeter and analyzes the fraction of expired oxygen using a galvanic fuel cell sensor. The REE was calculated by the system using a constant respiratory quotient (RQ) of 0.85, following the Weir equation, as per standard device methodology. Although this is a standard clinical configuration, we acknowledge that interventions that induce nutritional ketosis or prolonged fasting may shift substrate oxidation towards fatty acids. This would lower the physiological RQ. Consequently, a fixed RQ of 0.85 may slightly underestimate the absolute REE in these specific study arms. Nevertheless, this fixed RQ was applied uniformly to all participants and time points, ensuring methodological uniformity and preserving the validity of our between-group REE change comparisons. Participants were required to fast for 8–12 h prior to each measurement, in accordance with the study protocol, and compliance with this requirement was confirmed before calorimetry assessment, and participants were also instructed to avoid vigorous exercise for 24 h. Assessments were conducted in a quiet, thermoneutral room (22–24 °C) following a 10–15 min rest period in a supine position. Oxygen consumption (*VO_*_2_) was recorded for 15 min through a silicone face mask, with the first 5 min discarded to ensure a steady state, leaving a 10 min stabilization period for the final REE calculation. To minimize circadian variability, post-intervention measurements were performed at the same time of day as the baseline assessment. The device was calibrated daily according to manufacturer’s specifications [20,21].

### 2.4. BHB Quantification by 1H NMR Spectroscopy

Spectral data were obtained using a Bruker AVANCE™ 600 MHz NMR spectrometer (Bruker BioSpin, Ettlingen, Germany) coupled to an Avance III console. The spectra were collected with a nitrogen-cooled TCI Prodigy cryoprobe. The acquisition protocol comprised several pulse sequences to improve the metabolite characterization, including one-dimensional Nuclear Overhauser Effect Spectroscopy (1D NOESY), Carr–Purcell–Meiboom–Gill (CPMG) experiments for attenuation of macromolecular signals through T2 filtering, and two-dimensional J-resolved spectroscopy (JRES) to facilitate metabolite assignment. Quantification of BHB was performed from the CPMG spectra using Chenomx software (v10.0, Chenomx Inc., Edmonton, AB, Canada). The absolute concentrations were determined using the Electronic Reference To Access In vivo Concentrations (ERETIC) method, implemented in TopSpin 3.5.pl7 (ERETIC 2), which served as the quantitative reference signal.

### 2.5. Statistical Analysis

The statistical analyses were performed using Jamovi software (Version 2.5) based on the R environment (Version 4.3) and using the GAMLj module (Version 3.0). The descriptive statistics for continuous variables are presented as the median and interquartile range (IQR) and the categorical variables are presented as frequencies and percentages. The normality of data distribution and model residuals were verified using the Shapiro–Wilk test, and homogeneity of variances was assessed using Levene’s test.

To evaluate the differences in REE among the five dietary groups, a Linear Mixed Model (LMM) was employed. This approach was selected to account for the hierarchical structure of the data (repeated measures nested within individuals) and to maximize the use of the available sample (n = 102 at baseline). An LMM is robust to data missing at random, allowing for the inclusion of 98 participants (150 total observations) in the final longitudinal model. The model included the intervention group, time (baseline vs. 3 months), and their interaction (Group × Time) as fixed effects. To explore the REE trajectories while accounting for potential biological correlates of energy expenditure, the model was adjusted for age, sex, total body weight, and beta-hydroxybutyrate (BHB) levels as fixed covariates, to control for any potential baseline inter-individual variability. To avoid multicollinearity, the total body weight and fat-free mass (FFM) were not included in the same model due to their high mathematical and biological correlation. Instead, a sensitivity LMM was performed by substituting the total body weight with the FFM to specifically evaluate the contribution of metabolically active tissue while avoiding collinearity with total body weight. A random intercept for participant ID was included to account for inter-individual variability. Degrees of freedom were estimated using the Satterthwaite approximation. The model fit was evaluated using the marginal *R*^2^ (variance explained by fixed effects) and conditional *R*^2^ (total variance explained by the model) as global effect sizes. To evaluate the practical and clinical relevance of the findings, the primary measure of effect size for individual predictors and interactions was reported as unstandardized regression coefficients (β), expressed in absolute units (kcal/day), along with their 95% confidence intervals (CIs). Prior to the formal modeling, a preliminary descriptive analysis of unadjusted absolute changes in REE was performed using a one-way ANOVA. The Estimated Marginal Means (EMMs) were calculated from the LMM to display the adjusted differences between groups and timepoints. To strictly control for Type I errors across the multiple study arms, post hoc pairwise comparisons of the EMMs were adjusted using a Bonferroni correction.

All analyses were two-sided, and a *p*-value < 0.05 was considered statistically significant. Figures were generated using the Matplotlib (Version 3.9) and Seaborn (Version 0.13) libraries in a Python environment (Version 3.12). The graphical abstract presented in this manuscript was assembled and designed using Canva. A specific graphical element representing the indirect calorimetry was generated using the artificial intelligence tool Nano Banana Pro (Gemini 3 Pro architecture; Google LLC, Mountain View, CA, USA). The authors manually integrated, edited, and annotated this AI-generated element along with other standardized graphics within Canva (Canva Pty Ltd., Sydney, Australia). All visual components were thoroughly reviewed by the authors to ensure strict scientific accuracy and proper representation of the study’s findings.

## 3. Results

### 3.1. Baseline Characteristics

**Figure 1:**
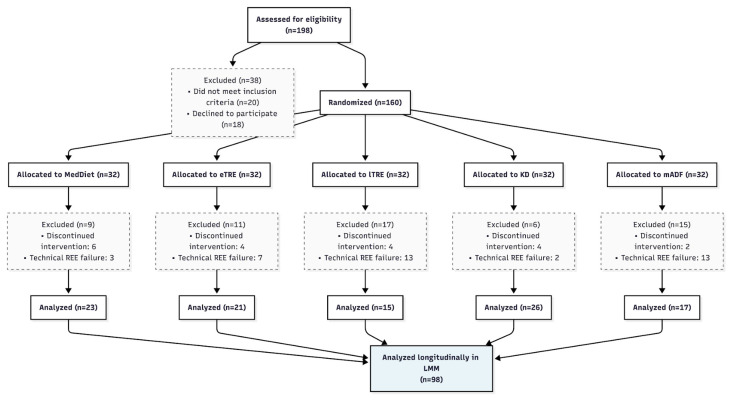
CONSORT flow-chart of study participants. Abbreviations: MedDiet, Mediterranean diet (control); eTRE, early time-restricted eating; lTRE, late time-restricted Eating; KD, ketogenic Diet; mADF, modified alternate-day fasting.

A total of 102 participants with valid baseline measures for REE were initially included. While a complete paired dataset (both baseline and 3-month follow-up) was available for 59 participants, the primary LMM framework accommodated missing-at-random observations without case deletion. This allowed for the longitudinal analysis of 98 participants (representing 150 total observations), significantly maximizing data retention and sample representativeness. The 58 participants from the original randomized cohort for whom calorimetry data were unavailable were excluded because of technical equipment failures or clinical scheduling conflicts. As described in the Methods Section, these administrative exclusions were not related to intervention adherence, weight loss outcomes, or predefined metabolic variables. The study flowchart is shown in Figure 1.

Baseline characteristics of the study population are presented in Table 1. The groups were homogeneous at baseline regarding age, anthropometric measures and REE, with no significant differences observed between the dietary interventions prior to the start of the study. To ensure the representativeness of the sub-sample, a comparative analysis of baseline characteristics and final weight loss between the included and excluded participants was performed (Supplementary Table S1). No significant differences in primary REE determinants, such as age, baseline weight or sex distribution, were found. While a slight difference was observed in baseline BMI (38.5 vs. 37 kg/m^2^, *p* = 0.039), the clinical relevance of this difference was marginal. Furthermore, the weight loss and body composition changes at 3 months were comparable between those included and excluded from this sub-analysis, supporting the representativeness of this sub-sample.

### 3.2. Descriptive Comparison of Unadjusted REE Changes

**Figure 2:**
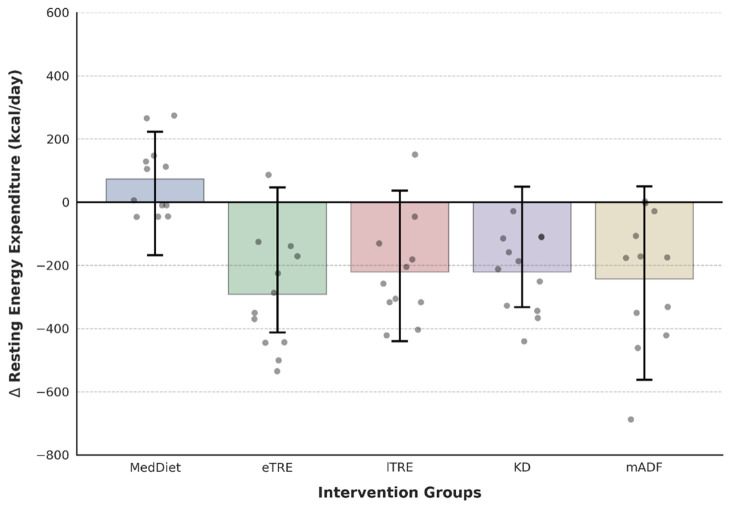
Unadjusted absolute changes in resting energy expenditure (REE) across dietary intervention groups. Data are presented as mean change (kcal/day) from baseline to end of intervention (n = 59 participants with complete paired longitudinal data). Individual data points are overlaid with horizontal jitter to illustrate data distribution and variability. Error bars represent 95% confidence intervals. One-way ANOVA indicates no statistically significant differences in unadjusted REE mean change among five groups (*p* = 0.382). Abbreviations: MedDiet, Mediterranean diet (control); eTRE, early time-restricted eating; lTRE, late time-restricted eating; KD, ketogenic diet; mADF, modified alternate-Day fasting.

First, the descriptive changes in REE from the baseline to the end of the intervention were evaluated. The unadjusted data from participants who completed both calorimetry assessments (n = 59) revealed a raw metabolic decline in all intervention groups, except for the MedDiet group, which maintained relative stability (mean change: 27.3; 95% CI: −168 to 223 kcal/day). The largest absolute reductions were observed in the mADF (−256; 95% CI: −562 to 49.7 kcal/day) and lTRE (−202; 95% CI: −440 to 36.2 kcal/day) groups. However, a one-way ANOVA confirmed that these raw differences in the REE change were not statistically significant among the five dietary interventions (*p* = 0.382) (Figure 2). These unadjusted results provided the rationale for the subsequent longitudinal modeling to account for baseline variability and key metabolic covariates.

### 3.3. Linear Mixed Model Analysis of REE Trajectories

To evaluate the impact of the dietary interventions on the REE while accounting for the longitudinal nature of the data and baseline variability, an LMM was employed. Of the total 102 participants included, the longitudinal analysis was based on the 98 participants who provided sufficient observations for the model, minimizing the bias from missing values at follow-up.

The model showed high explanatory power (conditional *R*^2^ = 0.718). The primary outcome, the interaction between intervention group and time, was not statistically significant (F(4,75.1) = 0.838; *p* = 0.506), indicating that the REE trajectory over the 3-month period did not differ significantly among the five dietary groups (Table 2).

In this comprehensive model, the total body weight (*p* < 0.001) and biological sex (*p* < 0.001) were the only significant independent predictors of REE. Notably, changes in circulating BHB (*p* = 0.275) did not independently influence the REE trajectory, suggesting that no independent association between BHB levels and REE trajectories could be detected within this model.

To address the potential issue of multicollinearity between total body weight and FFM, a sensitivity LMM analysis was performed by substituting the total body weight with the FFM (Supplementary Table S2). While the FFM emerged as a significant independent predictor of REE (*p* < 0.001), this secondary model exhibited a lower overall explanatory power (conditional *R*^2^ = 0.636). In this sensitivity analysis, the mADF group showed a nominally significant association with lower REE values compared with the MedDiet group (*p* = 0.045). However, given the exploratory nature of this sub-analysis, the reduced explanatory power of the model, and the multiple comparisons performed, this isolated finding should be interpreted cautiously. Overall, the sensitivity analysis supported the general consistency of the primary findings, suggesting that the REE trajectories were more strongly associated with the baseline biological characteristics (sex, age, FFM) than with the specific dietary strategy employed.

### 3.4. Estimated Marginal Means

**Figure 3:**
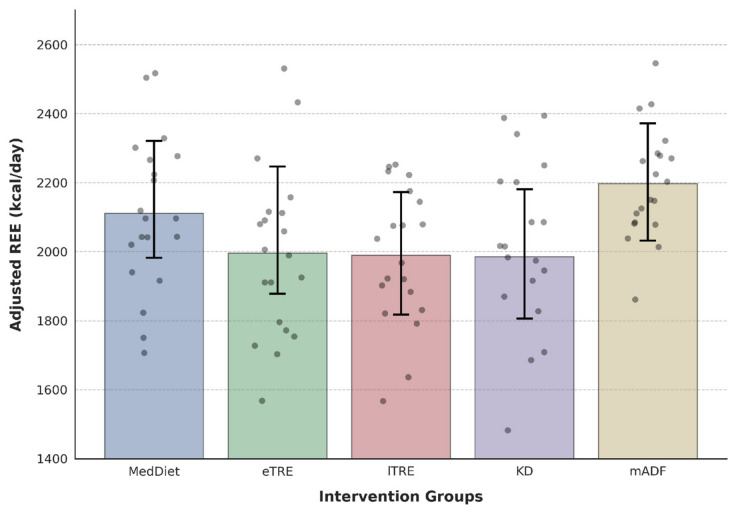
Adjusted REE at 3 months across intervention groups. Data represent adjusted Estimated Marginal Means derived from Linear Mixed Model, accounting for baseline age, sex, body weight, and beta-hydroxybutyrate (BHB) levels. Bars represent adjusted means. Individual data points are overlaid with horizontal jitter to ensure readability and transparency regarding individual variability. Error bars represent 95% confidence intervals calculated from original model output. No significant differences are observed across groups (Group × Time interaction, *p* = 0.506).

The Estimated Marginal Means (EMMs) were calculated to represent the REE trajectories adjusted for all model covariates (age, sex, weight, and BHB levels). Consistent with the primary LMM findings, no statistically significant differences were observed in the adjusted REE trajectories among the five intervention groups (Group × Time interaction, *p* = 0.506). Although numerical trends were noted, specifically a tendency toward a higher adjusted REE in the mADF group (2202 kcal/day) compared to the KD group (1993 kcal/day) at the end of the intervention, the overlap of the 95% confidence intervals and the non-significant interaction term confirm the lack of differences in the REE in our sample after these dietary strategies. These findings suggest that no independent association between dietary strategy and REE trajectories could be detected within this model (Table 3, Figure 3).

## 4. Discussion

This sub-analysis was conducted as part of an RCT in patients with obesity undergoing weight loss. The main objective was to compare the effect of different hypocaloric dietary approaches, differing in their theoretical ketogenic potential, on the variation in REE measured via indirect calorimetry. The principal finding of this sub-analysis is that no statistically significant differences in the trajectory of REE could be reliably detected among the five dietary patterns in this dataset, regardless of the macronutrient composition or meal timing. To maximize data retention and account for the longitudinal nature of the study, a Linear Mixed Model (LMM) was employed, confirming that neither the KD nor the intermittent fasting strategies (eTRE, lTRE, and mADF) demonstrated detectable superior metabolic advantages over the hypocaloric MedDiet within the statistical power of this exploratory sub-analysis when adjusting for key biological covariates and potential mediators. These results complement our primary analysis, in which we observed that while the KD, mADF, and lTRE were more effective for absolute weight loss than the MedDiet, these differences did not appear to be driven by the preservation of REE. The robust fit of the LMM supports the consistency of these observations within this dataset. However, it is important to emphasize that the relatively wide confidence intervals observed across several group comparisons suggest limited statistical precision.

Our findings are consistent with those of Heilbronn et al. [22] and Catenacci et al. [23], who reported no detectable changes in REE following fasting, supporting the hypothesis that temporal eating restrictions do not substantially alter basal expenditure. In analyses focusing primarily on REE, no additional detectable change was observed in response to complete alternate-day fasting. Conversely, Soeters et al. [24] suggested a significant decrease in REE of 59 kcal/day with IF compared to a standard diet after 2 weeks, and Sutton et al. [25] suggested that energy expenditure remained unaffected by time-restricted feeding, based on the absence of significant differences in body mass compared to a standard diet, despite the absence of objective energy expenditure measurements. In addition, regarding KDs, our observation of no significant REE preservation is aligned with the findings of Hall et al., who noted that while substantial carbohydrate restriction significantly impacts substrate oxidation, it does not result in a clinically significant or sustained increase in energy expenditure, as measured in a metabolic chamber, when compared to isocaloric standard diets [11]. The discrepancies often seen in the outpatient literature favoring KDs are increasingly attributed to isotopic artifacts inherent to the doubly labeled water method rather than true thermogenic advantages. Similarly, regarding IF, the MATADOR trial [17], using indirect calorimetry, showed that alternating periods of severe restriction with isoenergetic maintenance could significantly attenuate the drop in REE by periodically relieving the continuous energy deficit signal.

The observed decline in REE following weight loss is a well-documented physiological response to energy restriction. While often discussed under the framework of adaptive thermogenesis, a biological process to preserve energy stores, our data show that this reduction was proportional across all groups. In our longitudinal model, body weight (*p* < 0.001) and biological sex (*p* < 0.001) emerge as the strongest independent associations of post-intervention REE. Specifically, our analysis suggests persistent sex-related differences in REE, with females exhibiting a significantly lower REE (321 kcal/day less) than males, even after adjusting for total body weight and FFM. This raises the possibility that an organism’s REE may reflect an individual metabolic trajectory rather than a fixed “set-point” or calibration value for preserving energy stores. Previous evidence suggests that diet-induced energy depletion may be calibrated against this pre-existing value; thus, after the 12 weeks, the participants’ metabolisms may have been mostly influenced by their baseline metabolic characteristics rather than the specific nutritional strategy employed [26]. Therefore, our findings highlight the importance of understanding the individualized nature of REE changes to help prevent weight regain [27].

Regarding our initial hypothesis, we anticipated differences in the preservation of REE among the groups. During conventional CR, hormonal responses, such as a marked decline in leptin, act as sensors of energy deficit, signaling the brain to reduce expenditure [28,29]. Although the ketogenic state has been proposed as a countermeasure, potentially conferring an advantage of 100 to 300 kcal/day due to the cost of gluconeogenesis and the thermic effect of protein [9,29], our findings suggest that any potential metabolic advantage was not large enough to be detected as a significant preservation of REE within the statistical power of this sub-sample. In the LMM, changes in beta-hydroxybutyrate (BHB) levels did not predict the REE trajectory, and the KD group did not show superior maintenance of REE compared to the other interventions. This aligns with other clinical studies concluding that, after longer periods, there are no significant differences in total energy expenditure when comparing KDs with simple CR [29,30]. Beyond caloric benefits, the literature suggests that BHB acts as a critical signaling molecule. While extending beyond the direct measurements performed in the present study, theoretical models propose that BHB may inhibit histone deacetylase (HDAC); activate oxidative stress resistance genes, such as FOXO3A and SOD2; and block the NLRP3 inflammasome, which theoretically protects mitochondrial function and could enhance metabolic efficiency [29]. In parallel, recent integrative conceptual frameworks also emphasize the dynamic interaction between energy availability, substrate utilization, and nutrient timing, suggesting that chrono-nutritional strategies may influence metabolic adaptation through coordinated physiological responses beyond caloric restriction alone [31]. Nevertheless, these mechanistic concepts should be interpreted as broader physiological hypotheses derived from the literature rather than as direct mechanistic implications of the present dataset, since BHB signaling, HDAC activity, FOXO3A activation, inflammasome regulation, and mitochondrial function were not directly assessed in this study.

It is essential to integrate these findings with the results of the primary analysis of this trial, in which it was observed that a KD did not preserve FFM significantly more effectively than the other interventions [18]. To assess the independent role of body composition on REE, we performed a sensitivity analysis separating the total body weight and FFM to avoid multicollinearity. LMM analysis showed that the model including total body weight had a slightly higher overall explanatory power than the model including FFM (conditional R^2^ = 0.718 vs. 0.636). When total body weight was replaced by FFM in the sensitivity model, the FFM emerged as a highly significant predictor of REE trajectory (*p* < 0.001); however, there were no significant differences among the five dietary protocols. This suggests that during the active phase of weight loss, weight may exert a more detectable influence on the REE trajectory than subtle variations in lean mass, and that changes in REE depend not only on the quantity of metabolically active tissue but also on the optimization of cellular and mitochondrial efficiency, which may adapt similarly regardless of the energy substrate or feeding protocol utilized. This interpretation aligns with evidence suggesting that the response to an energy deficit is a highly individual and stable trait [32]. While some strategies in our trial, such as mADF and lTRE, showed a numerical trend toward a slightly higher adjusted expenditure, this did not reach statistical significance. From a physiological standpoint, these numerical trends are very interesting. It may be possible that the alternating structure of mADF intermittently alleviates the continuous energy deficit signal, while the late temporal window of lTRE may interact differently with circadian substrate oxidation patterns [17]. However, given our statistical constraints, these observations remain a speculative interpretation. Our data support the hypothesis that the ultimate impact on the REE depends more on the accumulated deficit and the individual’s individual physiological response than on the specific fasting protocol [33,34].

From a clinical perspective, these results emphasize that an energy deficit remains the primary driver of weight loss, and no specific dietary pattern evaluated here demonstrated intrinsic metabolic superiority regarding the REE outcomes, at least within the detection limits and the exploratory nature of this study [35,36]. Consequently, if the hypocaloric MedDiet, KD, and IF yield similar metabolic results, their long-term sustainability and patient preference become the critical determinants of success.

Finally, this study has limitations. As this study was a secondary sub-analysis, no specific a priori power calculation was performed for resting energy expenditure. The sample size analyzed in the longitudinal model (n = 98) was determined by the availability of valid longitudinal calorimetry measurements. This relatively small sample size and the resulting wide confidence intervals may have limited the ability to detect small but clinically meaningful differences (risk of Type II error). Therefore, the absence of statistically significant differences should be interpreted as an absence of detectable differences within the present dataset, rather than as evidence of true equivalence between dietary strategies. In addition, as this was a secondary sub-analysis, the risk of selection bias was rigorously assessed. A comparative analysis revealed no significant differences in baseline characteristics or final weight loss between the included and excluded participants (see Supplementary Table S1). This finding suggests that our sub-sample was broadly representative. We also acknowledge a technical limitation regarding our equipment, which assumed a fixed respiratory quotient (0.85). As detailed in the Methods Section, while this is a common clinical practice, actual shifts in substrate oxidation during ketogenic or fasting regimens might have led to minor variations in the absolute REE precision. Specifically, a fixed RQ of 0.85 may have slightly underestimated the true energy expenditure in individuals achieving deep nutritional ketosis. Although this represents a limitation, the use of a fixed RQ was applied consistently across all participants and time points, thereby maintaining the comparability of between-group differences in REE changes. As the primary objective of this sub-analysis was to compare the changes in REE between dietary groups, the consistent application of the same methodological approach across all participants supports the validity of these relative comparisons. Furthermore, the use of Linear Mixed Models provided a robust statistical framework to handle missing data and account for inter-individual variability, ensuring that the interpretation of the dietary effect was not biased by listwise deletion or over-adjustment of baseline variances.

## 5. Conclusions

In summary, this exploratory sub-analysis did not identify statistically significant differences in the preservation of REE among five different hypocaloric dietary patterns over a 12-week period. Our findings suggest that the changes in REE trajectories did not statistically diverge across the dietary strategies within the limits of this exploratory dataset, and no distinct effects of macronutrient composition or meal timing were detectable in the short term. The REE trajectories were more strongly associated with biological features, such as body weight and sex. Neither the specific diet type, final FFM, nor ketosis levels were significant factors in determining REE maintenance. This may be indicative of the potential impact of pre-existing metabolic characteristics on energy expenditure responses, rather than the specific nutritional strategies employed. The use of longitudinal modeling reinforced these findings by maximizing sample retention and providing a clear distinction between dietary effects and individual biological variance. While strategies such as KD, mADF, and lTRE were more effective for absolute weight loss in the primary trial, these clinical differences do not appear to be driven by a significant preservation of REE detectable within this dataset. Given the modest sample size and the risk of Type II error, these results should be interpreted as a lack of detectable differences rather than definitive proof of metabolic equivalence. These findings remain hypothesis generating and require long-term, primary-powered trials for confirmation, which could ultimately help to improve personalized care and prevent weight regain in obesity management.

## Figures and Tables

**Table 1:** Baseline characteristics of the study participants by dietary group.

Characteristic	MedDiet (n = 23)	eTRE (n = 21)	lTRE (n = 15)	KD (n = 26)	mADF (n = 17)	*p*
Sex (%) Females/Males	64/36	79.2/20.8	84.2/15.8	80/20	57.1/42.9	0.189
Age (years)	48.0 [42.0–56.0]	44.0 [35.0–51.5]	47.5 [43.0–54.8]	46.5 [39.5–52.8]	46.0 [42.8–51.0]	0.749
Weight (kg)	110.0 [96.7–127.0]	104.0 [94.5–114.0]	105.0 [94.4–113.0]	104.0 [89.0–114.0]	122.0 [106.0–130.0]	0.080
BMI (kg/m^2^)	38.7 [34.7–44.1]	37.3 [34.9–43.1]	38.2 [35.4–42.8]	38.1 [33.0–39.6]	42.2 [35.6–45.4]	0.167
Fat Mass (kg)	47.3 [38.0–53.0]	47.9 [40.4–54.3]	43.9 [36.6–52.9]	45.4 [33.3–54.3]	51.0 [43.5–59.6]	0.224
Fat-Free Mass (kg)	64.1 [54.1–77.9]	55.6 [51.4–64.1]	56.2 [51.6–67.8]	58.6 [52.6–61.5]	70.4 [52.8–78.4]	0.185
Baseline BHB (μM)	29.8 [17.8–42]	28.4 [23.3–50.2]	36.4 [29.8–47.4]	35.5 [28.8–50.1]	37.7 [32.1–66.7]	0.390
Baseline REE (kcal/day)	2017 [1842–2256]	2042 [1712–2410]	2176 [1718–2314]	2004 [1656–2208]	2349 [2112–2799]	0.075

Data are presented as median [interquartile range, Q1–Q3]. Abbreviations: BMI: body mass index; REE: resting energy expenditure; BHB: beta-hydroxybutyrate; MedDiet: Mediterranean diet; eTRE: early time-restricted eating; lTRE: late time-restricted eating; mADF: modified alternate-day fasting.

**Table 2:** Estimated coefficients from the Linear Mixed Model evaluating changes in resting energy expenditure at 3 months by intervention group, adjusted for age, sex, body weight, and BHB levels.

Variable	Coefficient (Beta), 95% CI	*p*-Value
**Intervention vs. Control**		
eTRE	−181.3 (−457.4, 94.8)	0.202
lTRE	−210 (−489.8, 69.7)	0.146
KD	−160.2 (−432.1, 111.6)	0.251
mADF	−217.5 (−486.6, 51.5)	0.117
**Covariates**		
Age (per year)	−6.4 (−12.9, −0.05)	0.051
Sex (female)	−313.3 (−465.3, −161.3)	<0.001
Body weight (kg)	12.4 (8.9, 16)	<0.001
BHB (µM)	−0.2 (−0.5, 0.1)	0.275

Values are expressed as regression coefficients with 95% confidence intervals (kcal/day). The model is adjusted for age, sex, body weight, and baseline BHB levels. Statistical significance is defined as *p* < 0.05. KD, ketogenic diet; eTRE, early time-restricted eating; lTRE, late time-restricted eating; mADF, modified alternate-day fasting; BHB, beta-hydroxybutyrate. The control group is the Mediterranean diet. The bold was used to separate the main outcome of the covariates and to make the table easier to understand.

**Table 3:** Estimated Marginal Means (EMMs) for resting energy expenditure (REE) across dietary groups at baseline and 3 months.

Group	Time	Mean	95% Confidence Interval
MedDiet	Baseline	2020	1869–2170
	3 months	2151	1982–2321
eTRE	Baseline	2112	1952–2272
	3 months	2063	1878–2247
lTRE	Baseline	2074	1881–2267
	3 months	1996	1818–2174
KD	Baseline	2022	1869–2175
	3 months	1993	1806–2181
mADF	Baseline	2287	2116–2459
	3 months	2202	2032–2372

Data are presented as mean and 95% confidence intervals (CIs) (kcal/day). Means are derived from Linear Mixed Model (LMM) and are adjusted for baseline age, sex, body weight, and beta-hydroxybutyrate (BHB) levels. No significant Group × Time interaction is observed (*p* = 0.506). MedDiet: Mediterranean diet; eTRE: early time-restricted eating; lTRE: late time-restricted eating; KD: ketogenic diet; mADF: modified alternate-day fasting.

## Data Availability

The dataset is available upon reasonable request to the corresponding author. The protocol is available on clinicaltrials.gov, and an extended version of the protocol is available upon reasonable request to the corresponding author.

## Supplementary Materials

The following supporting information can be downloaded at: https://www.mdpi.com/article/10.3390/nu18132053/s1, Table S1: Comparison of baseline characteristics between included and excluded participants for the REE sub-analysis; Table S2: Sensitivity Analysis. Estimated coefficients from the sensitivity linear mixed model evaluating changes in resting energy expenditure at 3 months by intervention group, adjusted for age, sex, fat-free mass, and BHB levels.

## Abbreviations

The following abbreviations are used in this manuscript:

BHB	Beta-hydroxybutyrate
BMI	Body mass index
CPMG	Carr–Purcell–Meiboom–Gill
CR	Caloric restriction
EMMs	Estimated marginal means
ERETIC	Electronic reference to access in vivo concentrations
eTRE	Early time-restricted eating
FM	Fat mass
FFM	Fat-free mass
FOXO3A	Forkhead box O3a
HDAC	Histone deacetylase
IF	Intermittent fasting
JRES	J-resolved spectroscopy
KD	Ketogenic diet
lTRE	Late time-restricted eating
MedDiet	Mediterranean diet
mADF	Modified alternate-day fasting
NLRP3	NOD-like receptor protein 3
REE	Resting energy expenditure
SOD2	Superoxide dismutase 2
TRE	Time-restricted eating
1D NOESY	One-dimensional nuclear Overhauser effect spectroscopy
