# Supplementary material for: Changes in Resting Energy Expenditure in Response to Different Dietary Patterns: A Randomized Clinical Trial Exploratory Sub-Analysis
Source: Nutrients. 2026 Jun 24;18(13):2053. doi: 10.3390/nu18132053 (PMC13363438; doi:10.3390/nu18132053)
Supplement: Supplementary file 1 [file nutrients-18-02053-s001.zip › nutrients-4359291-supplementary.pdf]

Supplementary Table S1. Comparison of baseline characteristics between included and excluded participants for the REE sub-analysis.

Characteristic	Included (n=102)	Excluded (n=58)	p-value
**Age (years)**	46.0 [42.0 – 51.0]	46.0 [38.0 – 56.0]	0.306[a]
**Weight (kg)**	107.3 [94.6 – 122.0]	103.5 [88.8 – 116.0]	0.210[a]
**BMI (kg/m²)**	38.5 [34.6 – 43.6]	37 [32.9 – 40.5]	**0.039[a]**
**Sex (Females, %)**	76 (74.5%)	37 (63.8%)	0.153[b]
**Weight loss at 3 months (kg)**	10.9 [7.7 – 15.4]	9.05 [6.5 – 12]	0.074[a]
**BMI change at 3 months (kg/m²)**	3.95 [2.78 – 5.37]	3.29 [2.29 – 4.68]	0.085[a]
**Fat mass change at 3 months (kg)**	8.45 [4.54-12.1]	6.25 [4.40-9.15]	0.084[a]

Data are presented as Median [Interquartile Range] for continuous variables and as n(%) for categorical variables. n: number of participants. BMI: Body Mass Index. [a]p-value calculated using the Mann-Whitney U test. [b]p-value calculated using the Pearson's Chi-squared test. **Bold** indicates statistical significance ($p < 0.05$). The 58 participants not included in the indirect calorimetry sub-analysis were excluded due to technical equipment failure or scheduling constraints, and not due to lack of adherence or clinical outcomes.

Supplementary Table S2. Sensitivity Analysis. Estimated coefficients from the sensitivity linear mixed model evaluating changes in resting energy expenditure at 3 months by intervention group, adjusted for age, sex, fat-free mass, and BHB levels.

Variable	Coefficient (beta) 95% CI	P-value
**Intervention vs. Control**		
eTRE	-269.6 (-580.1, 41.0)	0.094
lTRE	-192.2 (-506.8, 122.4)	0.236
KD	-201.3 (-505.7, 103.1)	0.199
mADF	-321.1 (-629.5, -12.8)	0.045
**Covariates**		
Age (per year)	-9.1 (-16.1, -2.1)	0.013
Sex (female)	-346.1 (-523.8, -168.4)	< 0.001
Body fat-free mass (kg)	10.5 (5.2, 15.8)	< 0.001
BHB (μM)	-0.3 (-0.7, 0.2)	0.230

Values are expressed as regression coefficients with 95% confidence intervals (kcal/day) This sensitivity model substituted total body weight with fat-free mass (FFM) to evaluate model stability. Statistical significance was defined as $p < 0.05$. REE, resting energy expenditure; KD, ketogenic diet; eTRE, early time-restricted Eating; lTRE, late time-restricted eating; mADF, modified alternate-day fasting; BHB, beta-hydroxybutyrate. Control group is the Mediterranean diet.
